# Ion channel and biophysical properties of extracellular vesicles

**DOI:** 10.1016/j.jbc.2026.111364

**Published:** 2026-03-10

**Authors:** Shridhar Kiran Sanghvi, Harpreet Singh

**Affiliations:** 1 Department of Physiology and Cell Biology, College of Medicine, The Ohio State University Wexner Medical Center, Columbus, Ohio, USA; 2 Department of Molecular, Cellular and Developmental Biology, The Ohio State University, Columbus, Ohio, USA

**Keywords:** extracellular vesicles, ion channels, transporters, homeostasis, trafficking, cell signaling, signal transduction, therapeutics, endomembrane

## Abstract

Extracellular vesicles (EVs) are a heterogeneous population of lipid bilayer–enclosed particles secreted by nearly all cell types into the extracellular milieu. Once considered cellular debris, EVs are now recognized as biologically active entities capable of transferring proteins, lipids, and nucleic acids to recipient cells, thereby modulating their function and contributing to intercellular communication. EVs play pivotal roles in immune regulation, signal transduction, and antigen presentation. EV molecular cargo reflects the physiological or pathological state of the parent cell, offering potential as diagnostic and prognostic biomarkers in a range of diseases, including cancer, neurodegeneration, and cardiovascular disorders. Traditionally, EVs have been classified into exosomes, microvesicles, and apoptotic bodies based on their size and biogenesis. Recent discoveries have expanded this taxonomy to include novel subtypes with distinct biophysical and molecular characteristics. This review focuses on EVs, with an emphasis on their biogenesis, mechanisms of ionic balance and homeostasis, and the presence and function of ion channels and transporters. We also highlight current methodologies for detecting functional ion channels within exosomes, underscoring their emerging significance in cellular physiology and disease pathogenesis.

Extracellular vesicles (EVs) represent a heterogeneous collection of lipid bilayer vesicles that are secreted in the extracellular environment by most cell types. In the past decade, EV research has gained significant interest, driven by their emerging roles in intercellular communication, disease pathogenesis, and their potential as biomarkers and therapeutic vehicles. The first evidence for the presence of EVs was provided in the early 1980s (1), when EVs were initially thought to represent the “*shedding of specific membrane*” during cell turnover or damage, with little to no effect on surrounding cells. Recent studies have shown that EVs are biologically active vesicles that are capable of transporting diverse molecular cargo, including proteins (2), lipids (3), and nucleic acids (2, 4, 5, 6), to the recipient cells. This delivery of molecular cargo has been shown to influence and reprogram the function of the target cells. As a result, EVs have become important mediators of intercellular communication and have been shown to play a key role in processes, such as immune regulation (7), signal transduction (8), and antigen presentation (9). Since nearly all eukaryotic cells can secrete EVs, their content can vary widely depending on the cell type and its physiological or pathological state. Therefore, the molecular contents of EVs have the potential to serve as diagnostic or prognostic indicators in various conditions, including cancer (10), chronic inflammation (11), lipid metabolism disorders (12), neurodegenerative disorders (13), cardiovascular diseases (14) and kidney diseases (15).

EVs are broadly categorized based on their biogenesis and release mechanisms into subtypes, such as exosomes (50–150 nm) (16, 17, 18), microvesicles (150–1000 nm) (17, 19, 20) and apoptotic bodies (100–5000 nm) (21, 22, 23). In addition, EVs can be classified according to their cellular origin, for instance, plasma-, platelet-, or endothelial-derived vesicles. Similarly, EV classification can also be based on the physiological or pathological state of the parent cell, like “oncosomes” secreted by cancer cells and “prostasomes” originating from prostate tissue. While exosomes, microvesicles, and apoptotic bodies have traditionally been considered the principal EV subtypes, recent advances have expanded this classification to include novel entities, such as supermeres (∼30 nm) (24, 25), exomeres (30–50 nm) (26, 27), ectosomes (100–1000 nm) (28), large oncosomes (>1000 nm) (17, 29), migrasomes (500–3000 nm) (30), exophers (50–4000 nm) (17, 31) and membrane particles. These emerging subtypes exhibit distinct biophysical properties and molecular compositions, suggesting specialized roles in intercellular communication and disease pathogenesis.

In this review, we provide a concise overview of EVs focusing on their biogenesis and the mechanisms by which they maintain ionic balance and homeostasis. We also discuss the presence and roles of ion channels and transporters within EVs. Finally, we outline the biophysical properties of EV membranes and their implications for ion channels within EVs and EV-mediated regulation of ion channels in recipient cells, as well as their therapeutic implications.

## Biogenesis of EVs

The biogenesis of EVs is a complex process involving multiple pathways and components. Typically, EVs are created from late endosomes (LEs) *via* inward invagination of the multivesicular body membrane (MVB), thus forming intraluminal vesicles (ILVs), which are then released into the extracellular space (32). Throughout the EV biogenesis pathway, a plethora of protein complexes are known to play significant roles in creating an EV. EV biogenesis can be broadly classified into two primary pathways: the endosomal sorting complex required for transport (ESCRT)-dependent and ESCRT-independent pathways (33, 34). The ESCRT machinery is crucial for the budding of ILVs within MVBs, with key protein complexes like ESCRT 0, I, II, and III initiating the process of MVB formation, vesicle budding, and cargo sorting (33, 35). In contrast to ESCRT-dependent mechanisms, the ESCRT-independent pathway bypasses traditional ESCRT proteins and relies on ceramide metabolism, with sphingomyelin synthase 2 contributing to ceramide synthesis that facilitates EV biogenesis (36).

## ESCRT-dependent pathway

**Figure 1:**
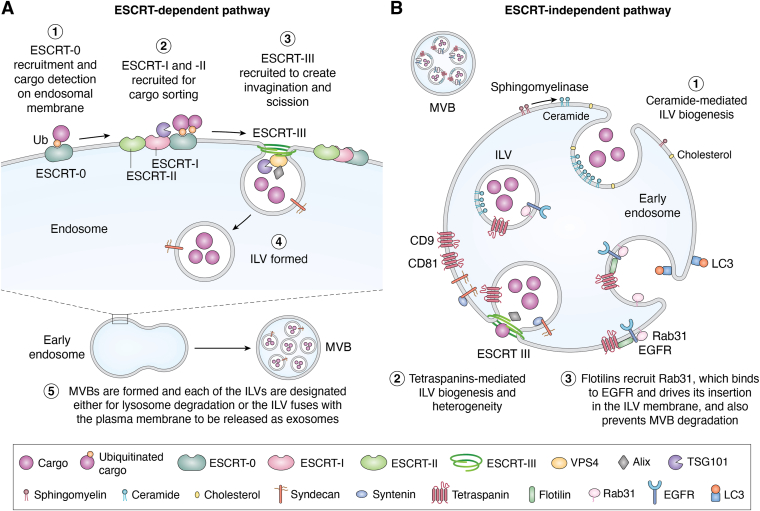
**Biogenesis of extracellular vesicles *via* ESCRT-dependent and ESCRT-independent pathways.** Multivesicular bodies (MVBs) form through two mechanisms. *A,* ESCRT-dependent pathway: ESCRT complexes (0–III) sequentially mediate cargo recognition, membrane invagination, and ILV scission, assisted by ALIX, TSG101, and VPS4. *B,* ESCRT-independent pathway: ILV formation is driven by ceramide, tetraspanins, and syndecan–syntenin complexes, with flotillins recruiting Rab31 to incorporate EGFR and prevent MVB degradation. Both pathways culminate in exosome release upon MVB fusion with the plasma membrane. EGFR, epidermal growth factor receptor; ESCRT, endosomal sorting complex required for transport; ILV, intraluminal vesicle.

ESCRT is a crucial component in cellular membrane trafficking, particularly in the formation of MVBs and the subsequent release of EVs. ESCRT proteins play an essential role in the sorting and transport of membrane proteins destined for degradation and in the biogenesis of EVs, which are significant players in intercellular communication and biomolecule transfer. The ESCRT machinery comprises several complexes designated as ESCRT-0, ESCRT-I, ESCRT-II, and ESCRT-III, each of which plays specific roles in the vesicle trafficking pathway. ESCRT-0 is primarily responsible for recognizing and binding ubiquitinated cargo at early endosomes. Following this initial step, ESCRT-I and ESCRT-II further facilitate the sorting of these cargo proteins into ILVs within MVBs (37, 38). Last, ESCRT-III allows the budding of ILVs from the limiting membrane of the endosome into its lumen (38, 39). The ESCRT complex operates by recognizing ubiquitinated proteins, which are tagged for degradation, binding to them, and escorting them into MVBs through a highly coordinated and conserved process. After the formation of ILVs, MVBs can either fuse with lysosomes for degradation or fuse with the plasma membrane to release EVs (40, 41). This selection of cargo is critical, as it determines the functional and molecular diversity of the resultant EVs, affecting their roles in physiological and pathological processes (41, 42). Furthermore, the EV protein ALIX, which is known to interact with various ESCRT components, such as TSG101 and CHMP4, has been shown to play a role in endosomal membrane budding, membrane scission, and the selection of EV cargo through its interaction with syndecan (Fig. 1*A*) (43).

## ESCRT-independent pathway

The EV biogenesis process relies on the ESCRT-independent pathway when ESCRT components are deficient or inactive to facilitate the formation and secretion of EVs. The ESCRT-independent EV biogenesis pathway primarily involves lipids and proteins that promote the budding and release of EVs without the involvement of the ESCRT machinery. A central mechanism in this pathway is the presence of ceramide, a sphingolipid derived from sphingomyelin *via* the action of neutral sphingomyelinase (19, 44). Ceramide promotes membrane curvature, which aids in the budding process of ILVs from the endosomal membrane and contributes to exosome formation (19, 45). In terms of cargo sorting within EVs, the tetraspanin family has emerged as a vital contributor to the ESCRT-independent pathway. Tetraspanins like CD9, CD63, and CD81 are involved in organizing lipid rafts and facilitating the clustering of membrane proteins, thus influencing the sorting and enrichment of specific cargo within EVs (44, 45). These aggregates may enrich certain signaling molecules or functional proteins, leading to heterogeneous EV populations based on their cellular origin (45, 46).

Rab GTPases also play a key role in the ESCRT-independent pathway, particularly in regulating membrane dynamics and vesicle transport. Rab31 has been identified as a key regulator in this pathway, controlling the secretion of EVs derived from ILVs independently of ESCRT components (47). Rab proteins are also integral to various stages of the EV life cycle, including vesicle fusion with the plasma membrane, where EVs are released. While the ESCRT-dependent pathway is more widely studied and understood because of its well-defined protein interactions, the ESCRT-independent pathway presents unique advantages in certain cellular contexts. This alternative pathway permits the continuous release of EVs even in the absence of a fully functional ESCRT system, which can be beneficial for cells under mechanical or environmental stress (45, 48). Moreover, it enables the production of EVs carrying distinct sets of cargo, influencing intercellular communication and contributing to processes, such as inflammation or tumor progression (Fig. 1*B*) (19, 44).

## Other factors governing EV biogenesis

Molecular-level modifications and interactions also substantially affect EV biogenesis. Post-translational modifications of proteins like SNAP23 and ALIX can impact the efficiency of EV formation and secretion (49). Alterations in O-GlcNAcylation have been shown to affect EV abundance, indicating that post-translational modifications serve as crucial regulatory checkpoints in EV pathways (49). A recent study has revealed the mechanistic role of syndecans and their associated partners in the production of EVs. Heparanase-mediated conditioning of syndecans has been demonstrated to activate the syndecan-syntenin-ALIX pathway, promoting EV formation enriched with specific cargo critical for cellular communication (35). The loading and sorting of EV cargo molecules remain a significant area of ongoing research, as they can determine the functional impact of EV signaling on recipient cells.

The microenvironment and external stimuli also modulate EV biogenesis. In pathological conditions like asthma, EV production and secretion correlate with inflammatory responses, suggesting that extracellular signals can drive EV biogenesis (50). Increased levels of proinflammatory cytokines, notably tumor necrosis factor-alpha, have been associated with heightened EV biogenesis, emphasizing the influence of environmental factors on cellular mechanisms involved in EV release (50). The cellular context influences the unique EV profiles based on the originating cell type. Variations in lipid composition, protein content, and RNA profiles significantly contribute to EV heterogeneity (51, 52). Thus, understanding the specific mechanisms driving EV formation across different cellular contexts is critical for understanding their contents and how they could potentially regulate the cellular processes, which will be crucial for leveraging their potential therapeutic applications.

## Biophysical properties of endomembranes

The electrophysiological properties of EVs are strongly influenced by their surface charge, which plays a critical role in colloidal stability, cellular uptake, and biodistribution. EVs typically exhibit a negative zeta potential (53), primarily because of the presence of anionic phospholipids, such as phosphatidylserine and phosphatidylinositol, on the outer leaflet of their lipid bilayer. EVs are generally derived from cellular membranes, yet their measured zeta potentials often differ from those of the parent cell surface (53, 54). This suggests that the physicochemical factors determining EV zeta potential remain elusive. In addition to anionic phospholipids, surface charges are also influenced by integral membrane proteins, glycosylation patterns, and the interaction of the outer leaflet with surrounding ions and charged biomolecules. Extracellular conditions, such as pH, ionic strength, and the presence of divalent cations (*e.g.*, Ca^2+^, magnesium [Mg^2+^]), can further modulate the electrostatic properties of EVs, influencing their aggregation behavior and interaction with the target cells (53). Insights from lysosome biophysics add another layer of complexity: lysosomes maintain an acidic lumen (pH ∼4.5–5.0) through V-ATPase-driven proton pumping, creating steep proton and ion gradients across their membranes, which influence their biophysical properties. Since EVs originate from the endolysosomal system, we predict that these gradients and associated membrane proteins can influence lipid composition and charge distribution, potentially affecting EV maturation, cargo sorting, and the electrostatic landscape of their surface.

**Figure 2:**
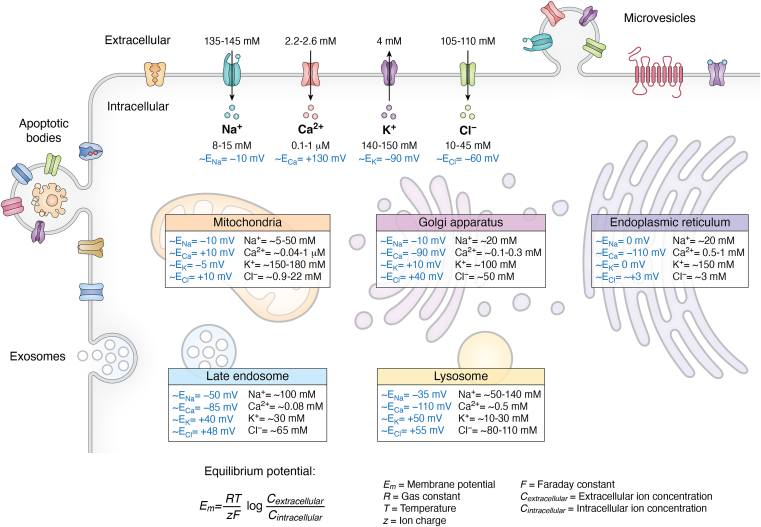
**Ionic composition and membrane potential across cellular organelles and extracellular vesicles.** This schematic compares the electrochemical environment of major intracellular compartments, emphasizing their distinct ionic gradients and membrane potentials. Equilibrium potentials (E_m_) for Na^+^, K^+^, Ca^2+^, and Cl^-^ are indicated for each compartment, reflecting the driving forces for ion movement and signaling. Endosomes and multivesicular bodies exhibit negative potentials with *high* K^+^ and *low* Ca^2+^, supporting vesicle maturation and trafficking. Extracellular vesicles maintain ionic conditions influenced by their origin, contributing to cargo loading and intercellular communication. These gradients are critical for organelle function, vesicle biogenesis, and signaling pathways.

Another key factor influencing the biophysical properties of EVs is ion fluxes across lipid membranes. Lipid bilayers are impermeable to ions, and the movement of ions occurs through various channels and transporters in the membrane (55). Since the flux of ions through the channels contributes to the membrane potential, we anticipate it potentially modulating surface properties of EVs. The ionic gradient across the membrane, which is maintained through energy-dependent processes (either *via* primary or secondary active transport), generates a driving force for the ions to move across the membrane (56). Hence, the membrane potential plays a crucial role in numerous cellular processes, such as signaling mechanisms, electrical signaling, cellular excitability, cell proliferation, survival, and differentiation. Under physiological conditions, the intracellular Na^+^ concentration is ∼14 mM, whereas the extracellular Na^+^ concentration is ∼140 mM, and the calculated equilibrium potential (E) E_Na_ is +60 mV. For K^+^, the intracellular concentration is ∼140 mM, and the extracellular concentration is ∼4 mM; hence, the E_K_ is −90 mV. The intracellular Ca^2+^ concentration is ∼100 nM, and the extracellular concentration is ∼2.2 mM, which gives it the highest E_Ca_ of +130 mV. For chloride (Cl^-^), the intracellular concentration ranges from 10 to 30 mM and extracellular concentration is ∼110 mM, with E_Cl_ ranging from −30 mV to −60 mV (Fig. 2). Since EVs are released in the extracellular environment, the presence of primary or secondary active transport is crucial for their survival (57).

Similar to the plasma membrane, intracellular organelle membranes maintain distinct ionic microenvironments essential for their specialized functions. These gradients are tightly regulated by intracellular organelle-specific ion channels, transporters, and pumps. The endoplasmic reticulum (ER) acts as a major Ca^2+^ reservoir, with luminal Ca^2+^ concentrations ranging from ∼0.5 to 1 mM, supporting roles in calcium signaling and protein folding (58, 59). The ER also maintains high K^+^ (∼150 mM) (60) and comparable Na^+^ (<20 mM) concentrations, resulting in an E_K_ near 0 mV and E_Na_ also around 0 mV (assuming a cytosolic [Na^+^] of ∼15 mM) (57, 61). However, the counteranion Cl^-^ in the ER is around ∼3 mM, with an E_Cl_ of −30 mV. The Golgi apparatus shares similar ionic features, with Ca^2+^ concentrations of 0.1 to 0.3 mM (E_Ca_ of −90 mV) and K^+^ ∼100 mM (E_K_ of 10 mV). It also maintains Na^+^ ∼20 mM with E_Na_ of ∼-10 mV and moderate Cl^-^ (∼50 mM) levels, with an overall E_Cl_ of approximately 40 mV (57, 62).

In the mitochondrial matrix, ion concentrations are tightly regulated to support bioenergetic and signaling functions. K^+^ levels are maintained at ∼150 to 180 mM for osmotic balance and volume regulation (63). Ca^2+^ concentrations range from ∼40 nM at rest to ∼1 μM during stimulation, enabling transient buffering and signaling (64). Cl^-^ levels vary between 0.9 and 22 mM, depending on metabolic state and channel activity (65), whereas Na^+^ concentrations range from ∼5 to 50 mM, influenced by mitochondrial Na^+^/Ca^2+^ and Na^+^/H^+^ exchangers (66). LEs exhibit a markedly different ionic profile, with low K^+^ (∼30 mM), high Na^+^ (∼51 mM), and an acidic pH (5.5–6.3). These conditions yield a strongly positive K^+^ (E_K_ +40 mV) and a positive Na^+^ (E_Na_ ∼-30 mV), favoring Na^+^ influx (57, 67). Cl^-^ concentrations are moderate to high (∼70 mM) with E_Cl_ of 50 mV, contributing to osmotic balance and charge compensation during H^+^ pumping by the V-ATPase (68, 69, 70, 71).

Another determinant that influences the biophysical properties of the endomembrane is membrane fluidity and lipid packing (72). The ER is characterized by loose lipid packing because of its enrichment in monounsaturated lipids, which contrasts with the tighter packing observed in plasma membranes rich in saturated lipids and cholesterol (73, 74). This gradient of membrane order across the secretory pathway has functional implications for protein folding and ion channel trafficking. Lipid regulation of ion channels is categorized as (a) direct lipid–protein interactions: lipids bind to specific sites on ion channels, influencing gating and activity and (b) indirect modulation *via* membrane properties: lipids alter membrane stiffness and elasticity, which in turn affects channel function (75). Thus, we posit that the origin of EVs from biomembranes will influence the expression of ion channels and their functions.

## Ion channels and transporters

Ca^2+^ plays a crucial role in a wide range of cellular processes (76, 77). Acting as a universal second messenger, Ca^2+^ influences various cellular functions, such as cytoskeletal movement, enzyme activity regulated by phosphorylation and dephosphorylation, and the secretion of molecules like neurotransmitters. Similarly, several studies have explored the mechanisms linking elevated intracellular Ca^2+^ concentrations to increased EV production (78, 79, 80). Vesicle trafficking is heavily dependent on Ca^2+^, since Ca^2+^ is integral to the processes of vesicle budding and fusion within the donor cells. Elevated intracellular Ca^2+^ levels are known to facilitate membrane fusion events essential for the release of EVs (81, 82, 83). Furthermore, these studies attribute an increase in exocytosis *via* Ca^2+^-dependent interactions with soluble *N*-ethylmaleimide-sensitive factor attachment receptor proteins and phospholipids. This indicates that changes in Ca^2+^ concentration directly influence the formation and release of vesicles containing various biological cargo, including proteins and nucleic acids (78, 79, 81).

**Figure 3:**
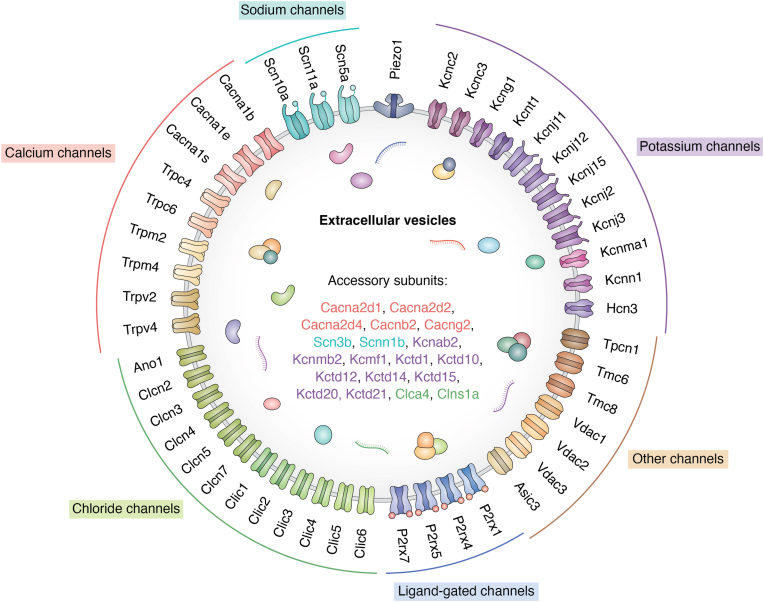
**Ion channel and accessory subunits detected in extracellular vesicles (EVs).** Schematic representation of the ion channels identified on EV membranes into functional groups based on ion selectivity: calcium channels (including voltage-gated Ca^2+^ channels and TRP family members), sodium channels, potassium channels (voltage-gated, inwardly rectifying, and/or calcium-activated), chloride channels (voltage-sensitive and/or calcium-activated), ligand-gated channels (ATP-gated P2X receptors), mechanosensitive channels (Piezo1), and other channels (two-pore channels, transmembrane channel-like proteins, and voltage-dependent anion channels). Associated modulatory subunits are categorized according to their function within the channel (inside). The subcellular localization of each channel or subunit indicates its predominant position within mammalian cells. Localizations include plasma membrane (Ano1, Asic3, Nav, and Kv families), presynaptic or postsynaptic membranes (CACNA1B, CACNG2), endosomal/lysosomal compartments (ClC-3/4/5/7, TPCN1), mitochondrial outer membrane (VDAC family), and ER/Golgi regions (TMC6/8, CLIC family).

Electrical characterization of EVs has revealed voltage-dependent and pH-sensitive currents (84). This behavior is consistent with the presence of a lipid bilayer membrane, similar to that of cells, which incorporates various membrane-associated proteins (85). Among these proteins, several function as ion channels, facilitating ion transport across the EV membrane (Fig. 3) (86). This explains the observed pH-dependent conductivity of EVs, which may result from altered ion transport dynamics through these membrane channels. In addition, Na^+^ transporters have also been identified in EV membranes. Na^+^/H^+^ exchanger 3 has been detected in urinary EVs and is considered a potential biomarker for kidney injury because of its critical role in Na^+^ reabsorption and fluid balance in renal physiology (87). Similarly, several ion transporters and exchangers have been reported on EV membranes (Table 1). We speculate that these ion channels and transporters help maintain ionic balance by mediating the controlled movement of ions across EV membranes, thereby preserving electrochemical gradients essential for EV volume regulation, pH homeostasis, and physiological signaling.

**Table 1:** Comprehensive list of membrane transporters and regulatory proteins detected in EVs

No.	Gene name (transporters)	Gene symbol	Function
1	Arsenite transporter, ATP-binding, homolog 1	ASNA1	Transporter
2	ATPase, Na+/K+ transporting, alpha 1 polypeptide	ATP1A1	Transporter
3	ATPase, Na+/K+ transporting, alpha 2 polypeptide	ATP1A2	Transporter
4	ATPase, Na+/K+ transporting, alpha 3 polypeptide	ATP1A3	Transporter
5	ATPase, Na+/K+ transporting, beta 1 polypeptide	ATP1B1	Transporter
6	ATPase, H+/K+ exchanging, alpha polypeptide	ATP4A	Transporter
7	ATP synthase, H+ transporting, mitochondrial F1 complex, alpha subunit 1, cardiac muscle	ATP5A1	Transporter
8	ATP synthase, H+ transporting, mitochondrial F1 complex, beta polypeptide	ATP5B	Transporter
9	ATP synthase, H+ transporting, mitochondrial Fo complex, subunit E	ATP5I	Transporter
10	ATP synthase, H+ transporting, mitochondrial Fo complex, subunit G	ATP5L	Transporter
11	ATP synthase, H+ transporting, mitochondrial F1 complex, subunit O	ATP5O	Transporter
12	ATPase, H+ transporting, lysosomal accessory protein 2	ATP6AP2	Regulator of transporters
13	ATPase, H+ transporting, lysosomal 70 kDa, V1 subunit A	ATP6V1A	Transporter
14	ATPase, H+ transporting, lysosomal 42 kDa, V1 subunit C2	ATP6V1C2	Transporter
15	Cystinosin, lysosomal cystine transporter	CTNS	Transporter
16	Ecto-NOX disulfide-thiol exchanger 1	ENOX1	Other (nontransporter)
17	FXYD domain–containing ion transport regulator 2	FXYD2	Regulator of transporters
18	Increased sodium tolerance 1 homolog	IST1	Other (nontransporter)
19	Magnesium transporter 1	MAGT1	Transporter
20	Mitochondrial calcium uniporter	MCU	Transporter
21	Membrane magnesium transporter 1	MMGT1	Transporter
22	Solute carrier family 10 (sodium/bile acid cotransporter), member 1	SLC10A1	Transporter
23	Solute carrier family 10 (sodium/bile acid cotransporter), member 1	SLC10A1	Transporter
24	Solute carrier family 12 (sodium/potassium/chloride transporter), member 1	SLC12A1	Transporter
25	Solute carrier family 12 (sodium/potassium/chloride transporter), member 2	SLC12A2	Transporter
26	Solute carrier family 12 (sodium/chloride transporter), member 3	SLC12A3	Transporter
27	Solute carrier family 12 (potassium/chloride transporter), member 4	SLC12A4	Transporter
28	Solute carrier family 12 (potassium/chloride transporter), member 5	SLC12A5	Transporter
29	Solute carrier family 12 (potassium/chloride transporter), member 6	SLC12A6	Transporter
30	Solute carrier family 12 (potassium/chloride transporter), member 7	SLC12A7	Transporter
31	Solute carrier family 13 (sodium-dependent dicarboxylate transporter), member 2	SLC13A2	Transporter
32	Solute carrier family 13 (sodium-dependent dicarboxylate transporter), member 3	SLC13A3	Transporter
33	Solute carrier family 15 (oligopeptide transporter), member 2	SLC15A2	Transporter
34	Solute carrier family 16 (monocarboxylate transporter), member 1	SLC16A1	Transporter
35	Solute carrier family 1 (neuronal/epithelial high affinity glutamate transporter, system Xag), member 1	SLC1A1	Transporter
36	Solute carrier family 1 (glial high affinity glutamate transporter), member 3	SLC1A3	Transporter
37	Solute carrier family 1 (neutral amino acid transporter), member 5	SLC1A5	Transporter
38	kidney specific organic anion transporter	SLC21A4	Transporter
39	Solute carrier family 22 (organic cation transporter), member	2SLC22A2	Transporter
40	Solute carrier family 22 (organic anion/urate transporter), member 11	SLC22A11	Transporter
41	Solute carrier family 22 (organic anion/urate transporter), member 12	SLC22A12	Transporter
42	Solute carrier family 22 (organic anion/urate transporter), member 13	SLC22A13	Transporter
43	Solute carrier family 22 (organic cation transporter), member 2	SLC22A2	Transporter
44	Solute carrier family 22 (organic cation/carnitine transporter), member 5	SLC22A5	Transporter
45	Solute carrier family 22 (organic anion transporter), member 6	SLC22A6	Transporter
46	Solute carrier family 22 (organic anion transporter), member 8	SLC22A8	Transporter
47	Solute carrier family 24 (sodium/potassium/calcium exchanger), member 2	SLC24A2	Transporter
48	Solute carrier family 25 (mitochondrial carrier; citrate transporter), member 1	SLC25A1	Transporter
49	Solute carrier family 25 (mitochondrial carrier; phosphate carrier), member 3	SLC25A3	Transporter
50	Solute carrier family 25 (mitochondrial iron transporter), member 37	SLC25A37	Transporter
51	Solute carrier family 26 (anion exchanger), member 11	SLC26A11	Transporter
52	Solute carrier family 26 (anion exchanger), member 2	SLC26A2	Transporter
53	Solute carrier family 26 (anion exchanger), member 4	SLC26A4	Transporter
54	Solute carrier family 26 (anion exchanger), member 6	SLC26A6	Transporter
55	Solute carrier family 26 (anion exchanger), member 8	SLC26A8	Transporter
56	Solute carrier family 26 (anion exchanger), member 9	SLC26A9	Transporter
57	Solute carrier family 29 (equilibrative nucleoside transporter), member 1	SLC29A1	Transporter
58	Solute carrier family 2 (facilitated glucose transporter), member 1	SLC2A1	Transporter
59	Solute carrier family 2 (facilitated glucose transporter), member 12	SLC2A12	Transporter
60	Solute carrier family 2 (facilitated glucose transporter), member 14	SLC2A14	Transporter
61	Solute carrier family 2 (facilitated glucose transporter), member 2	SLC2A2	Transporter
62	Solute carrier family 2 (facilitated glucose transporter), member 3	SLC2A3	Transporter
63	Solute carrier family 2 (facilitated glucose transporter), member 4	SLC2A4	Transporter
64	Solute carrier family 2 (facilitated glucose/fructose transporter), member 5	SLC2A5	Transporter
65	Solute carrier family 34 (type II sodium/phosphate cotransporter), member 1	SLC34A1	Transporter
66	Solute carrier family 34 (type II sodium/phosphate cotransporter), member 2	SLC34A2	Transporter
67	Solute carrier family 35 (UDP-GlcNAc/UDP-glucose transporter), member D2	SLC35D2	Transporter
68	Solute carrier family 36 (proton/amino acid symporter), member 1	SLC36A1	Transporter
69	Solute carrier family 36 (proton/amino acid symporter), member 2	SLC36A2	Transporter
70	Solute carrier family 37 (glucose-6-phosphate transporter), member 2	SLC37A2	Transporter
71	Solute carrier family 3 (amino acid transporter heavy chain), member 1	SLC3A1	Regulator of transporters
72	Solute carrier family 3 (amino acid transporter heavy chain), member 2	SLC3A2	Regulator of transporters
73	Solute carrier family 40 (iron-regulated transporter), member 1	SLC40A1	Transporter
74	Solute carrier family 44 (choline transporter), member 2	SLC44A2	Transporter
75	Solute carrier family 4 (anion exchanger), member 1 (Diego blood group)	SLC4A1	Transporter
76	Solute carrier family 4, sodium borate transporter, member 11	SLC4A11	Transporter
77	Solute carrier family 4 (anion exchanger), member 2	SLC4A2	Transporter
78	Solute carrier family 4 (sodium bicarbonate cotransporter), member 4	SLC4A4	Transporter
79	Solute carrier family 4, sodium bicarbonate cotransporter, member 7	SLC4A7	Transporter
80	Solute carrier family 4, sodium bicarbonate cotransporter, member 8	SLC4A8	Transporter
81	Solute carrier family 5 (sodium/glucose cotransporter), member 1	SLC5A1	Transporter
82	Solute carrier family 5 (sodium/sugar cotransporter), member 10	SLC5A10	Transporter
83	Solute carrier family 5 (sodium/monocarboxylate cotransporter), member 12	SLC5A12	Transporter
84	Solute carrier family 5 (sodium/glucose cotransporter), member 2	SLC5A2	Transporter
85	Solute carrier family 5 (sodium/myoinositol cotransporter), member 3	SLC5A3	Transporter
86	Solute carrier family 5 (sodium/iodide cotransporter), member 5	SLC5A5	Transporter
87	Solute carrier family 5 (sodium/multivitamin and iodide cotransporter), member 6	SLC5A6	Transporter
88	Solute carrier family 5 (sodium/monocarboxylate cotransporter), member 8	SLC5A8	Transporter
89	Solute carrier family 5 (sodium/sugar cotransporter), member 9	SLC5A9	Transporter
90	Solute carrier family 6 (neurotransmitter transporter), member 13	SLC6A13	Transporter
91	Solute carrier family 6 (amino acid transporter), member 14	SLC6A14	Transporter
92	Solute carrier family 7 (anionic amino acid transporter light chain, xc- system), member 11	SLC7A11	Transporter
93	Solute carrier family 7 (amino acid transporter light chain, L system), member 5	SLC7A5	Transporter
94	Solute carrier family 8 (sodium/calcium exchanger), member 1	SLC8A1	Transporter
95	Solute carrier family 9, subfamily A (NHE1, cation proton antiporter 1), member 1	SLC9A1	Transporter
96	Solute carrier family 9, subfamily A (NHE3, cation proton antiporter 3), member 3	SLC9A3	Transporter
97	Solute carrier family 9, subfamily A (NHE3, cation proton antiporter 3), member 3 regulator 1	SLC9A3R1	Regulator of transporters
98	Solute carrier family 9, subfamily A (NHE3, cation proton antiporter 3), member 3 regulator 2	SLC9A3R2	Regulator of transporters
99	Solute carrier family 9, subfamily A (NHE4, cation proton antiporter 4), member 4	SLC9A4	Transporter
100	Solute carrier family 9 (sodium/hydrogen exchanger), member 8	SLC9A8	Transporter
101	Solute carrier family 9, subfamily A (NHE9, cation proton antiporter 9), member 9	SLC9A9	Transporter
102	Solute carrier organic anion transporter family, member 1a1	SLCO1A1	Transporter
103	Solute carrier organic anion transporter family, member 1A2	SLCO1A2	Transporter
104	Solute carrier organic anion transporter family, member 3A1	SLCO3A1	Transporter
105	Solute carrier organic anion transporter family, member 4A1	SLCO4A1	Transporter
106	Solute carrier organic anion transporter family, member 4C1	SLCO4C1	Transporter

Ion channels in EVs contribute to the regulation of their ionic composition, structural stability, and the controlled release of biomolecular cargo (88). Notably, the functional activity of large-conductance calcium-activated and voltage-gated potassium (BK_Ca_) channels, encoded by the *Kcnma1* gene, has been recorded in plasma-derived EVs from mice. BK_Ca_ channels exhibited iberiotoxin-sensitive single-channel conductance of ∼335 pS, confirming the presence of functional BK_Ca_ channels. Furthermore, BK_Ca_ channels are essential for maintaining the structural and functional integrity of EVs and play a key role in selective cargo packaging. In EVs isolated from *Kcnma1*^−^^/^^−^ mice, a small K^+^ conductance of ∼100 pS was also observed, which may be attributed to alternative K^+^ channels, as suggested by data from the ExoCarta protein database (88).

Furthermore, engineered mesenchymal stem cell (MSC)-derived EVs expressing a cystic fibrosis transmembrane conductance regulator (CFTR) zinc finger fusion protein with transcriptional activation domains were shown to activate CFTR transcription and restore Cl^-^ transport in human bronchial epithelial cells (89). The presence of EVs in a range of sizes suggests a role for volume regulation, potentially mediated by Cl^-^ channels. Proteomic analyses have identified several Cl^-^ channels in EVs, implicating them in volume regulation; however, functional characterization of these channels is necessary to elucidate their role in determining EV size and cargo composition. Another Cl^-^ channel, chloride intracellular channel 4 (CLIC4), has been identified in circulating EVs released by breast cancer cells. CLIC4-containing EVs influence the dynamic interactions between tumor cells and CLIC4-expressing stromal cells, which help shape the EV cargo profile. This coordinated exchange may contribute to the development of a supportive premetastatic niche. CLIC4 is consistently detectable in EVs from both tumor-bearing mice and human breast cancer patients. This finding implicates CLIC4 as a potential biomarker, although its pathological significance remains to be fully elucidated (90).

The ability of EV channels and transporters, coupled with their diverse cargo of proteins, RNA, and other biomolecules, positions them as crucial players in various biological processes, including cancer progression, neuronal signaling, and tissue regeneration (91, 92, 93). The channel activity in EVs could potentially regulate the cargo content within the EVs (88), rendering them important tools for understanding disease mechanisms and identifying potential therapeutic targets, supporting their growing importance in biomedical research.

## Regulation of ion channels through EV content

EVs act as dynamic signaling entities, delivering molecular cargo that modulates ion channel activity through direct interactions, intracellular signaling pathways, and post-transcriptional regulation (94, 95, 96). This functional regulation of ion channels implies a broader physiological role of EV biophysics, linking structural properties to intercellular communication. Over the past decade, numerous modulators of ion channels and transporters have been identified within EV cargo, suggesting that EVs may influence cellular behavior and physiological processes by directly or indirectly regulating ion channel activity. One direct mechanism involves connexins, which can form functional channels between EV membranes and recipient cells. Proteomic analyses have identified connexin isoforms, such as Cx43, Cx45, and Cx32, in EVs derived from various cell types (97, 98, 99). These connexins facilitate the transfer of ions and signaling molecules, thereby modulating the functional state of ion channels in target cells (100). Notably, Cx43 has been shown to form functional hemichannels on EV surfaces, enabling the release of cargo, such as luciferin, into recipient cells. Furthermore, the release of cargo has been shown to be inhibited by Cx43-specific blockers (101). The widespread secretion of Cx43-containing EVs across diverse cell types suggests a conserved communication mechanism, potentially governed by connexin subtype and tissue-specific expression. Therapeutically, Cx43-enriched vesicles have demonstrated promise in targeted drug delivery applications, such as doxorubicin transport, by enhancing delivery specificity and minimizing off-target cardiotoxicity (102).

Another prominent mechanism by which EVs influence ion channel function is through the transfer of signaling molecules and regulatory proteins. The circulating EVs from remote ischemic preconditioned mouse hearts exhibit elevated levels of miR-144, which is associated with increased phosphorylation of activation of protein kinase B (AKT), glycogen synthase kinase 3β, and p44/42 mitogen-activated protein kinase (103). Similarly, EVs derived from Epstein–Barr virus–infected nasopharyngeal carcinoma cells contain latent membrane protein 1, which activates extracellular signal-regulated kinase and AKT signaling pathways in recipient cells (104). These signaling cascades are known to modulate ion channel activity. In neurons, AKT signaling enhances L-type Ca^2+^ channel activity *via* insulin-like growth factor-1 and phosphorylates the voltage-gated sodium channel Nav1.1, reducing peak Na^+^ currents (105). In cardiomyocytes, PI3K and its downstream effector AKT regulate multiple ion currents, including L-type calcium, rapid and slow delayed rectifier potassium, and Na^+^ currents (106). These findings underscore the capacity of EV content to modulate ion channel function through complex intracellular signaling networks.

Beyond protein-mediated mechanisms, EV cargo also includes a diverse array of noncoding RNAs, particularly miRNAs, which play a pivotal role in post-transcriptional gene regulation (107). miRNAs modulate ion channel expression by targeting specific mRNAs, thereby influencing physiological processes, such as neuronal excitability, cardiac electrophysiology, and cellular homeostasis. The delivery of miRNAs *via* EVs enables the modulation of both intrinsic and recipient cell ion channel activity, integrating into broader regulatory networks (107, 108).

A key mechanism of miRNA-mediated regulation involves the targeted repression of ion channel–encoding mRNAs. This regulation can induce phenotypic changes aligned with the physiological roles of the affected ion channels (109). In mesial temporal lobe epilepsy, miRNA-ion channel interactions significantly influence neuronal excitability and network dynamics (110). miR-129-5p suppresses Kv1.1 mRNA in neuronal dendrites (111), whereas miR-27a-3p inhibits the expression of voltage-gated potassium channels *KCNB1* and *KCNQ2*. In addition, downregulation of miR-155 *via* preoperative valproic acid administration has been shown to prevent postoperative seizures by upregulating *SCN1A* (112). A miR-324-5p-dependent mechanism has also been implicated in the regulation of K^+^ channel function and seizure susceptibility (113). Also, miR-145 and miR-494 have been shown to directly target CFTR mRNA, thereby modulating epithelial fluid and electrolyte transport (114, 115). These interactions illustrate the broader significance of miRNA-mediated regulation in maintaining homeostasis and contributing to disease pathogenesis.

Importantly, many of the miRNAs implicated in ion channel regulations have been identified within EV cargo (Table 2) (116). Thus, the regulation of ion channel activity by miRNAs represents a multifaceted interplay involving direct mRNA targeting, feedback mechanisms that influence miRNA expression, and modulation of ion channel function across diverse physiological and pathological contexts. A deeper understanding of these interactions holds significant therapeutic potential for correcting ion channel dysfunctions associated with neurological, cardiovascular, and epithelial disorders.

**Table 2:** miRNA detected in EVs that regulates ion channel and its association in diseased pathophysiology

miRNA	Activity	Diseased pathophysiology	References
miR-133a/b	Modulates the expression of Kv4.2, Kir2.1, along with HCN2 and HCN4	Cardiac hypertrophy, myocardial fibrosis, and arrhythmias	(204, 205, 206)
miR-195	Directly targets expression of CACNB1, KCNJ2, and KCND3	Cardiac hypertrophy, arrhythmia, prostate cancer, NSCLC, IVDD	(207, 208)
miR-365	Regulates repolarizing ion channels such as IKs	Short-QT and long-QT syndromes and modulates cardiac arrhythmias	(209)
miR-211/222	Targets CACNA1C (L-type calcium channel) and KCNJ5 (inward rectifier potassium channel)	Cardiac hypertrophy, vascular remodeling, and multiple cancers	(210, 211)
miR-129-5p	Regulates expression of TRPM7 and modulates PI3K/AKT/mTOR and WNT/β-catenin pathways	Digestive cancers, neurodegenerative diseases, depression	(108, 212)
miR-324-5p	Regulates expression of Kv4.2	Epilepsy, cardiac disease, and various cancers	(213, 214)
miR-223-3p	Regulates expression of Kv4.2	Cardiovascular diseases, inflammation, and cancer	(215, 216)
miR-301a	Regulation of Kv4.2	Diabetes, IBD, colitis-associated cancer, and pancreatic/gastric cancers	(217, 218)
miR-155	Regulates the expression of CACNA1C	Atrial fibrillation, neuroinflammation, Alzheimer’s, Parkinson’s, MS, and stroke	(219, 220)
miR-30b	Regulates expression of the sodium channel	Gastric, hepatic, cardiovascular diseases	(221, 222, 223)
miR-9	Regulates expression of anoctamin 1 and Nav1.1/Nav1.2 trafficking	Epilepsy and glioma-associated epilepsy	(224, 225)
miR-7a	Modulates VDAC1 and voltage-gated sodium channel β2 subunit	Alzheimer’s, encephalitis, cardiomyocyte apoptosis	(226, 227, 228)
miR-137	Regulates the expression of Cav1.3 and potassium-chloride cotransporter	Schizophrenia, glioma, and neurodevelopmental disorders	(229, 230)
miR-501-3p	Modulates glutamatergic transmission	Schizophrenia, ovarian and pancreatic cancer	(231)
miR-181a	Regulates GluA2 surface expression and glutamate ionotropic receptor AMPA type subunit 2 expression	Neurodegeneration and cardiovascular disease	(232, 233, 234)
miR-223-3p	Regulates Kv4.2 in cardiomyocytes	Cardiovascular diseases, inflammation, and cancer	(215, 216)
miR-124	Regulates CLIC1 and GRIN1	Stroke, spinal cord injury, and neurodegenerative diseases	(235, 236, 237)
miR-19	Regulates the expression of KCNE4 and GJA1	Atherosclerosis, endothelial dysfunction	(238, 239, 240)
miR-125b	Regulates the activity and expression of CFTR and ASIC1a expression	Myocardial ischemia, stroke, heart failure, and inflammation	(241, 242, 243)
miR-539	Targets EGFR, affects ion channel signaling	Breast and oral cancer	(244)

AMPA, α-amino-3-hydroxy-5-methyl-4-isoxazolepropionic acid; EGFR, epidermal growth factor receptor; IBD, inflammatory bowel disease; IVDD, intervertebral disc generation; MS, multiple sclerosis; NSCLC, non–small cell lung cancer; VDAC1, voltage-dependent anion channel 1.

## Methods to measure ion channel activity in EVs

Over the years, extensive tools have been discovered to measure the functional activity of ion channels. The tools range from planar bilayer, patch clamp, amperometry analysis, fluorescence-based assay, and flux-based tools. Hence, we have summarized a few methods that can be incorporated in understanding the channel activity of ion channels on EV membranes.

### Planar lipid bilayers

The earliest demonstration of ion channel activity in artificial biomolecular lipid films was achieved using lecithin or glycerol mono-oleate matrices incorporating gramicidin A. Under conditions of 1 M NaCl, these systems exhibited a conductance of approximately 2.4 × 10^-11^ Ω^-1,^ indicating that gramicidin A forms transient, ion-conductive pores rather than functioning as a simple ion carrier (117). Subsequently, the first successful incorporation of native vesicular membranes into artificial bilayers was demonstrated using fragmented sarcoplasmic reticulum vesicles derived from rabbit skeletal muscle. These vesicles were shown to fuse with black lipid membranes under defined experimental conditions, resulting in discrete, stepwise increases in membrane conductance, providing direct evidence of functional channel incorporation (118). Similarly, K^+^ currents were recorded for the first time in EV membranes using a planar lipid bilayer (PLB) (88), suggesting the presence of functional ion channels in EV membranes. This methodological advancement enabled the controlled study of native membrane proteins within PLB systems.

Over the past 5 decades, the PLB technique has remained a cornerstone in ion channel research because of its capacity to facilitate high-resolution, single-channel recordings under well-defined conditions. This approach allows for detailed molecular characterization of ion channel function, including pharmacological profiling using specific channel activators and inhibitors. The typical PLB setup consists of a dual-chamber system: *cis* and *trans* compartments, which are separated by a small aperture (typically 100–200 μm in diameter) across the lipid bilayer. On addition of membrane vesicles, ionic currents and membrane potentials can be measured in response to electrochemical gradients, yielding critical insights into ion conductance and channel gating mechanisms (119, 120).

When applying this technique to EV membranes, several experimental parameters must be carefully optimized. These include the lipid composition of the bilayer, the purity of the membranes/proteins, the ionic composition of the *cis* and *trans* solutions, the signal-to-noise ratio, and the overall versatility of the experimental setup. Such considerations are essential for achieving reliable and physiologically relevant recordings of ion channel activity from EV membranes.

### Patch clamp

The patch-clamp technique, pioneered by Erwin Neher and Bert Sakmann with their first recording of the single-channel currents recorded from the membrane of denervated frog muscle fibers using patch clamp (121), revolutionized electrophysiology by enabling the recording of ionic currents through individual ion channels in biological membranes. Traditionally, the patch-clamp technique was applied to intact cells, which involves the use of a glass micropipette to form a high-resistance seal with the cell membrane, allowing precise measurement of ion channel activity. Since the first invention of the patch-clamp system, several groups have used this method to record single-channel activity from endosomes, phagosomes, autophagosomes, lysosomes, mitochondria, chloroplasts, plant vacuoles, peroxisomes, and the nucleus (122, 123). However, this technique has its limitations in the size of the organelle that can be used for patching; EVs (<1 μm) are relatively smaller in size, making them suboptimal for patch-clamp studies. A significant advancement has been made where patch-clamp techniques are extrapolated for artificial systems, such as liposomes, for studying ion channel activity in a controlled environment. Hence, one of the alternative approaches using membrane-fused giant unilamellar vesicles (1–100 μm in diameter) or liposomes could potentially help identify native EV channels (124).

### Amperometric measurements

Actuators are unique amperometry sensors that measure ionic currents that are converted to charge over time. This method uses polypyrrole doped with dodecylbenzenesulfonate (PPy[DBS]), a redox-active conducting polymer, to detect localized ionic changes near biological tissues. The PPy(DBS) undergoes a reversible redox reaction, which facilitates ion exchange around the actuator. The detection relies on a working electrode, which is made of a platinum wire coated with PPy(DBS), and a chlorinated silver wire acting as both the counter electrode and reference electrode. These electrodes are kept at a fixed distance between them and equilibrated in the recording conditions. During sensing, the PPy(DBS) on the working electrode switches between oxidized and reduced states when the voltages are applied, and the acquired data are filtered at 10 to 20 Hz. Ion exchange at the working electrode will depend on the surrounding ion concentration. This sensing approach, called near-field electrophysiology, has been used to measure cationic fluxes from the neurons (125), the retrotrapezoid nucleus of neurons during the pH change (126), K^+^ channel activity in cancer cells (127), and tissue oxygen level in the striatum and hippocampus of rats (128). For the first time, this technique was applied to measure K^+^ currents in intact EVs. With the K^+^ actuator placed in the vicinity of EVs, the activity of iberiotoxin-sensitive BK_Ca_ channels was recorded in intact vesicles (88). Furthermore, near-field electrophysiology combined with mathematical modeling (129) and kinetics studies (130, 131) can estimate (i) the internal ion concentration of EVs and (ii) the number of channels per EV, and (iii) ion movement across the membrane in response to channel blockers, membrane disruption, or applied chemical gradients.

### Genetically encoded indicator–based ionic measurements

Genetically encoded indicators (GEIs) have transformed the study of cellular signaling by enabling real-time, noninvasive monitoring of intracellular ionic dynamics. These tools have proven particularly valuable for quantifying ion concentrations within specific subcellular compartments, offering insights into both physiological and pathophysiological processes. Among the most extensively developed GEIs are those for Ca^2+^ detection. Broadly, GEIs fall into two categories: (a) single-fluorophore-based sensors and (b) FRET-based sensors. Single-fluorophore GEIs, such as the GCaMP family (132, 133, 134), utilize a circularly permuted fluorescent protein fused to calmodulin (CaM) and a CaM-binding peptide. Upon Ca^2+^ binding, conformational changes in CaM induce chromophore deprotonation, resulting in increased fluorescence emission. The CaM domain contains four EF-hand motifs that coordinate Ca^2+^ binding, triggering structural rearrangements that enhance the interaction with the CaM-binding domain. FRET-based GEIs, in contrast, consist of a donor and acceptor fluorophore pair. Ca^2+^ binding induces a conformational change that reduces the distance between the fluorophores (<10 nm), facilitating nonradiative energy transfer. This alters the emission spectra of the donor and acceptor, enabling ratiometric quantification of Ca^2+^ levels (135).

Building on similar principles, genetically encoded potassium ion indicators and oxazine fluorescent K^+^ sensors have been developed to monitor intracellular and subcellular K^+^ gradients (136, 137, 138, 139). These sensors exhibit high specificity for K^+^ over other monovalent cations and can detect concentrations ranging from micromolar to millimolar levels (136, 138, 140). Genetically encoded zinc indicators, traditionally FRET based, are capable of detecting Zn^2+^ concentrations in the picomolar to nanomolar range (141, 142). More recently, robust single-fluorophore genetically encoded zinc indicators have been engineered for subcellular Zn^2+^ imaging (143, 144, 145). Mg^2+^ indicators are also primarily FRET based but exhibit crossreactivity with Ca^2+^. These sensors detect Mg^2+^ and Ca^2+^ in the millimolar range (146, 147, 148), making them suitable for studying divalent cation dynamics in organelles. Cl^-^-GEIs exploit the intrinsic Cl^-^ sensitivity of yellow fluorescent proteins, enabling detection of intracellular Cl^-^ concentrations in the 80 to 100 mM range (149, 150, 151). Ratiometric Cl^-^ sensors have been developed to measure concentrations from 8 to 30 mM, although their performance may be influenced by pH sensitivity or dissociation constants outside physiological Cl^-^ levels (152, 153, 154, 155).

To enable subcellular resolution, these GEIs are frequently engineered with organelle-targeting sequences or fused to resident organellar proteins, allowing precise localization to compartments, such as the mitochondria, ER, Golgi apparatus, and lysosomes (156, 157, 158). This spatial targeting enhances the ability to dissect compartment-specific ionic dynamics under physiological and pathological conditions. Furthermore, the application of GEIs is expanding into EV research as it provides a robust measurement (159, 160). Given their modular design and sensitivity, GEIs hold promise for characterizing the ionic composition of exosomes and other EVs, potentially offering new insights into intercellular communication and disease biomarkers.

## Therapeutic applications of EVs

### Cancer

EVs have gained substantial interest in cancer therapy because of their versatile roles in intercellular communication and their potential as therapeutic carriers. Notably, engineered exosomes have been reported to enhance the delivery of RNA interference drugs and chemotherapeutics directly to tumors, effectively mitigating the adverse effects commonly associated with traditional drug delivery methods (161, 162). The therapeutic applications of exosomes extend to their function as immunotherapeutic agents. Exosome-based immunotherapies are being developed to manipulate T-cell responses and augment antitumor immunity (163, 164). This strategy is supported by evidence demonstrating that exosomes can be designed to carry tumor-associated antigens, which trigger immune responses against cancer cells, thereby potentially revolutionizing the therapeutic landscape for cancer patients (164, 165).

EVs also influence the tumor microenvironment by affecting cellular processes like cell growth, invasion, and metastasis (166, 167). Furthermore, EVs can promote blood vessel formation and help create conditions for cancer to spread, making them both a target for therapy and a useful tool for monitoring disease progression (167). The ability of EVs to reflect the physiological and pathological state of tumors allows them to serve as promising biomarkers for diagnosis and prognosis in cancer (168). Engineering EVs for improved drug delivery further enhances their therapeutic potential. Recent advancements in EV modification techniques have shown that, through genetic and chemical functionalization, it is possible to improve the targeting precision and therapeutic efficacy of these natural nanocarriers (162, 169). A study showed an exosome-based delivery system for paclitaxel that effectively treats multidrug-resistant cancer cells (162), which reiterates EVs' potential for carrying other chemotherapeutic agents.

In addition, the biocompatibility and the ability of EVs to cross the blood–brain barrier (BBB) enhanced their significance in the treatment of brain metastases and other cancers previously deemed challenging to treat (170). EVs can effectively deliver therapeutics across such barriers, thus holding promise for therapies aimed at central nervous system (CNS) tumors (170, 171). Finally, combining EV-based treatments with existing therapies could improve outcomes by increasing effectiveness and ameliorating side effects (163).

### Cardiovascular diseases

Therapeutic application of EVs is gaining interest in cardiovascular medicine for their ability to support heart repair and regeneration after injuries like myocardial injury. Studies have demonstrated that EVs secreted by cardiac and MSCs harbor bioactive molecules that can promote cardiomyocyte survival, inhibit apoptosis, and stimulate angiogenesis (172, 173). EVs enriched with miRNAs, particularly those derived from human umbilical cord MSCs, have been shown to alleviate acute myocardial ischemia by promoting cell survival and neovascularization (174, 175). Such effects are crucial in protecting the myocardium from permanent damage and enhancing functional recovery postinfarction (174, 176).

EVs are also being explored as carriers for targeted drug delivery, especially for delivering cardioprotective agents or RNA-based therapies directly to heart tissue. Injecting exosomes into the myocardium can bypass systemic circulation and increase local drug concentration (177). However, challenges remain, such as the invasiveness of this approach and achieving precise cardiac targeting (177, 178). Interestingly, strategies like blocking the mononuclear phagocyte system can improve EV delivery to the heart, boosting the effectiveness of therapeutic molecules like miRNAs or proteins (178).

Beyond therapy, EVs play a key role in communication within the cardiovascular system. Under stress conditions like glucose deprivation or ischemia, cardiomyocytes release EVs that trigger angiogenic signals in endothelial cells (179). This helps restore blood flow and oxygen supply to damaged areas, supporting tissue survival (179). EVs also influence inflammation, remodeling, and fibrosis, shaping the onset and progression of cardiovascular disease. By transmitting regulatory signals, they affect fibroblast activation and endothelial function, underscoring their multifaceted role in heart health (180).

### Neurological diseases

The unique ability of EVs to mediate cellular communication and their capacity to cross the BBB and potential for delivering therapeutic cargo have shown promise for various conditions, including neurodegenerative diseases, strokes, and traumatic brain injuries (TBIs).

EVs act as mediators of intercellular communication in the CNS, carrying proteins, lipids, and RNAs that can influence neuronal development, repair, and survival. MSC-derived EVs have been shown to promote neurogenesis and enhance functional recovery in stroke models by delivering neuroprotective factors and promoting neurovascularization (181). These vesicles can also modulate inflammation and apoptosis, which are critical in various neurological disorders. The systemic administration of EVs has been shown to significantly improve outcomes in rodent models of ischemic stroke by reducing infarct size and enhancing neurological recovery (181). The therapeutic effects are attributed to the EV cargo that aids in cellular repair processes, suggesting the potential of EVs as novel treatment strategies for acute CNS injuries. In a clinical study, EVs derived from bone marrow stem cells demonstrated beneficial effects in amyotrophic lateral sclerosis models by alleviating neuronal degeneration and improving motor functions (182). In addition, engineered EVs, when loaded with specific cargo, such as small interfering RNAs or neuroprotective agents, have shown potential in modulating amyloid-beta accumulation and tau hyperphosphorylation (183, 184). In Alzheimer's disease, EVs can enhance the delivery of CB2 receptor agonists, providing a novel approach for reducing neuroinflammation, which is central to the pathology of the disease (185).

EVs are also emerging as a therapy for TBI. MSC-derived exosomes can limit cell death and support recovery by enhancing neuroprotection and repair (186). In animal studies, exosome treatment after TBI improved behavioral outcomes and reduced brain lesions (186, 187). Their ability to cross the BBB makes EVs an ideal candidate for brain-targeted therapies. Moreover, engineered EVs can be optimized for better brain delivery, enabling precise transport of therapeutic agents to the affected regions (183, 188).

EVs can cross the BBB, primarily through active transcytosis mechanisms rather than passive diffusion. Evidence from *in vitro* and *in vivo* studies suggests that uptake occurs *via* multiple endocytic pathways, including macropinocytosis (189, 190), clathrin-mediated endocytosis (189, 190), caveolae-mediated endocytosis (190), and adsorptive-mediated transcytosis (191). All the endocytosis pathways rely on the ionic dynamics for remodeling the membrane or the recruitment of the protein. While charge-based interactions are known to play a crucial role in endocytosis. The relevance of ion channels and transporters in mediating electrostatic interactions between the target membranes and EVs is still unknown.

### Immune diseases

One of the most promising applications in immune-related diseases is their ability to promote immune tolerance. MSC-derived EVs have demonstrated efficacy in suppressing inflammatory responses and promoting recovery in autoimmune diseases like experimental autoimmune encephalomyelitis and rheumatoid arthritis (192). The mechanisms of action involve the inhibition of Th17 cell differentiation and the lowering of the proinflammatory cytokines, thus shifting the immune environment toward tolerance (192).

In transplantation scenarios, EVs can help prevent graft rejection by encouraging immune tolerance. EVs derived from dendritic cells have been shown to improve immune regulation and support transplant success (193). EVs have also been explored for their ability to modulate allergic responses. EVs from dendritic cells treated with interleukin-10 can downregulate inflammatory TH2 responses associated with allergies, suggesting that these vesicles can promote a switch to a more regulatory immune environment (194).

In infectious diseases, EVs released by infected macrophages exhibit the capacity to activate the innate immune response, strengthening the defense against pathogens (195). Moreover, EVs derived from tumor cells contain a plethora of immunomodulatory molecules that can influence cancer progression. EVs containing Fas ligand and other immunosuppressive factors that downregulate T-cell responses, thus creating a protective niche for tumors (196, 197). Therefore, targeting these immunosuppressive EVs could boost antitumor immunity and enhance the effectiveness of cancer treatments.

### Other diseases

EVs derived from MSCs are increasingly recognized as promising therapeutic agents across a variety of diseases, beyond cancer or immune-related disorders. EVs derived from MSC demonstrate significant therapeutic potential in respiratory conditions, including asthma and pulmonary arterial hypertension. In preclinical models, MSC-EVs exhibit potent anti-inflammatory and immunomodulatory effects, capable of reducing airway remodeling and enhancing lung function (198). Emerging data indicate that MSC-EVs can precisely regulate inflammatory pathways in various lung diseases, positioning them as promising cell-free therapeutic candidates that extend beyond current conventional treatments (199).

EVs have emerged as promising tools in the field of regenerative medicine, particularly for wound healing. Studies have indicated that MSC-derived EVs can enhance the healing process in various tissue types, including skin and corneal injuries. The EVs' rich cargo of growth factors and cytokines promote cellular proliferation and migration, essential for repairing damaged tissues (200, 201).

## Conclusion and future directions

EVs have the potential to serve as vehicles for bioelectric signals, contributing to synchronized cellular responses in tissues where electrical activity is critical. This novel concept expands the functional repertoire of EVs beyond biochemical signaling. As stated earlier, EVs carry a net negative charge on their surface (zeta potential), which will influence their interaction with cell membranes or regulate channels *via* an electric field. In parallel, electric fields around cells can also influence the movement of EVs as well as fusion with target cells. The fusion of EVs can transiently alter the local membrane potential, which can have rapid consequences on the target cells. Theoretically charged EVs can be detected by field effect transistors. At the application level, altering the surface charge can assist in designing vesicles that can modulate cellular signaling, such as synaptic activity, arrhythmias, and in cancers.

Ionic homeostasis is a fundamental aspect of cellular physiology and plays a pivotal role in the biogenesis of EVs. The dynamic regulation of ion concentrations, such as Cl^-^, Ca^2+^, Na^+^, and K^+^, is critical during various stages of EV formation, from early intracellular assembly to vesicle release and functional activity. EVs are generated within LEs through inward budding of the endosomal membrane and thus are expected to exhibit an ionic composition reflective of the late endosomal lumen (49). In contrast, microvesicles, which originate by outward budding from the plasma membrane, are predicted to share the ionic profile of the cytosol (202). Similarly, apoptotic bodies, which arise from the fragmentation of apoptotic cells into membrane-bound subcellular components, are anticipated to retain cytoplasmic ionic concentrations. These vesicles may encapsulate intact organelles, such as mitochondria, nuclei, and lysosomes, each maintaining their characteristic ionic environments (203). EV biology has gained significant attention in the last decade because of its diverse functions, diagnostics, and therapeutic potential. However, little is known about the ionic homeostasis mediated by channels and transporters in these vesicles. Identification and understanding of ionic mechanisms will not only deepen our understanding of EV biology but also open exciting avenues for innovative diagnostic and therapeutic strategies, making this an exceptionally promising frontier in biomedical research.

## Data availability

All the raw data will be available upon request.

## Conflict of interest

The authors declare that they have no conflicts of interest with the contents of this article.
